# Milk-Related Symptoms and Immunoglobulin E Reactivity in Swedish Children from Early Life to Adolescence

**DOI:** 10.3390/nu10050651

**Published:** 2018-05-21

**Authors:** Jennifer L. P. Protudjer, Ola Olén, Mirja Vetander, Inger Kull, Erik Melén, Marianne van Hage, Magnus Wickman, Anna Bergström

**Affiliations:** 1 Center for Occupational and Environmental Medicine, Stockholm County Council, 113 65 Stockholm, Sweden; anna.bergstrom@ki.se; 2 Institute for Environmental Medicine, Karolinska Institutet, 171 11 Stockholm, Sweden; mirja.gotze-vetander@sll.se (M.V.); inger.kull@ki.se (I.K.); erik.melen@ki.se (E.M.); magnus.c.wickman@gmail.com (M.W.); 3 Clinical Epidemiology Unit, Department of Medicine Solna, Karolinska Institutet, 171 76 Stockholm, Sweden; Ola.Olen@ki.se; 4 Sachs’ Children and Youth Hospital, The South General Hospital, 118 83 Stockholm, Sweden; 5 Department of Clinical Science and Education, Sodersjukhuset, Karolinska Institute, 118 83 Stockholm, Sweden; 6 Department of Medicine Solna, Immunology and Allergy Unit, Karolinska Institutet, and Karolinska University Hospital, 171 76 Stockholm, Sweden; marianne.van.hage@ki.se; 7 Centre for Clinical Research Sörmland, Uppsala University, 751 85 Eskiltuna, Sweden

**Keywords:** adolescent, allergy, anaphylaxis, child, food hypersensitivity, Immunoglobulin E, milk

## Abstract

Cow’s milk often causes symptoms in infants. Whereas, some continue to experience symptoms through childhood, others become tolerant. Yet, the ages at which persistence and tolerance occur are less clear. Thus, we examined the age of onset and persistence of milk-related symptoms from early life to adolescence, and Immunoglobulin E (IgE) milk reactivity, focusing on gender differences in a large, population-based birth cohort. Overall, 20.0% (537/2985) of children, with a comparable gender distribution, had early life milk-related symptoms. At 16y, approximately 2% (62/2985) children had persistent symptoms and high milk IgE levels (e.g., median at 4 years: 1.5 kU_A_/L) that were beginning in early life. In contrast, 94% had transient symptoms and low median IgE levels (early life: 0.63 kU_A_/L, 8y: 0.72 kU_A_/L; 16 years: 1.1 kU_A_/L). Also, at 16 years, approximately 6% of females and 3% of males without any previously reported symptoms reported adolescent-onset of symptoms (*p* < 0.001). Such symptoms were almost exclusively gastrointestinal symptoms and were not associated with detectable IgE. In conclusion, early life milk-related symptoms are common, although most cases are transient by 16 years. Twice as many females vs. males report adolescent-onset symptoms, and particularly gastrointestinal symptoms. Children with persistent symptoms have both a higher prevalence and higher milk IgE levels, as compared to other phenotypes.

## 1. Introduction

Consumption of cow’s milk is common in many parts of the world. Yet, milk is also a common cause of food-associated symptoms in infancy [1,2]. Most, but not all, cases of milk-related symptoms in infancy are Immunoglobulin E (IgE) mediated [1,2,3]. Gastrointestinal and skin symptoms are often reported for both immunologically mediated and non-immunological phenotypes. Although more severe symptoms [4,5], including anaphylaxis [6], are rare, mild symptoms are common [2,3,4]. Further, affected children and their families report worse health-related quality of life [7,8] and increased household costs [9] than those who are tolerant to milk. Tolerance, which was defined as an absence of symptoms upon milk consumption, commonly occurs by school age [3]. However, some evidence suggests that tolerance occurs as late as adolescence [4], and/or to heated or baked milk, but not to milk as a beverage [3]. 

Only a couple of studies have prospective data on milk-related symptoms beyond the first decade of life, neither of which has taken gender into consideration or considered adolescent-onset symptoms [10,11]. As such, investigation is warranted both to confirm if tolerance is reported beyond late childhood, and to identify the characteristics, including gender, of adolescent-onset milk-related symptoms. To this end, we aimed to examine the age of onset and the persistence of cow’s milk-related symptoms from early life to adolescence, as well as IgE reactivity to milk, focusing on gender differences in the Swedish population-based birth cohort, named BAMSE.

## 2. Materials and Methods

### 2.1. Study Design

This study was based on data from BAMSE, a population-based, longitudinal, unselected birth cohort of 4089 children born in Stockholm, Sweden between 1994 and 1996 [12]. Pertinent to the present study, we used data from parent-completed questionnaires administered when the children were approximately two months old (baseline), and at ages 1, 2, 4, and 8 years. At age 16 years, data from both parent-completed and child-completed questionnaires were employed. At baseline, information on socio-economic status and parental allergy was collected. At ages 1, 2, 4, 8, and 16 years, we queried doctor-diagnosed food allergy, age of first introduction to milk, as well as symptoms to foods, and symptoms of asthma, eczema, and rhinitis. The response rate from baseline through age 16 years was 77.8% (3181/4089).

At age 16 years, participants completed a food frequency questionnaire, which included questions on milk and milk products (yogurt and sour milk, a common food in Sweden) in six pre-defined answers ranging from rarely or never, to at least daily). Herein, these answers were categorised as either less than weekly, or at least once weekly.

At ages 4, 8, and 16 years, all of the participants who answered the questionnaires were invited to a clinical follow-up visit, at which sera samples were taken. At these same ages, sera from 2077, 2109, and 2251 children, respectively, were analysed for IgE reactivity to a food allergen mix (ImmunoCAP fx5 (cow’s milk, egg white, codfish, peanut, soy bean, and wheat); Thermo Fisher/Phadia AB, Uppsala, Sweden), according to the manufacturer’s instruction. The results were expressed in kUA/L, and a positive test was defined as ≥0.35 kU_A_/L. Sera that scored ≥0.35 kU_A_/L to the food allergen mix, were further analysed for IgE reactivity to the specific foods in the mix, including milk. 

Our study population included children for whom information on milk-related symptoms (defined below) in early life, and at ages 8 and 16 years was available. This yielded our study population of 2985 (73.0% of the original cohort). We also defined a subpopulation of children for whom information on both milk-related symptoms and food allergens including milk IgE reactivity were available at ages 4, 8, and 16 years (*n* = 1634; 40.0% of the original cohort; 54.7% of the study population).

Permission was obtained by the Regional Ethical Review Board, Karolinska Institutet, Stockholm, Sweden.

### 2.2. Definitions

#### 2.2.1. Milk-Related Symptoms

At ages 1, 2, and 4 years (hereafter referred to as early life), and at ages 8 and 16 years, parents reported whether their children had experienced specific symptoms (vomiting, diarrhoea, eczema, urticaria, itch, facial oedema (not queried at age 8 years), oral allergy syndrome [13], and runny nose and/or wheeze), following milk consumption in the 12 months prior to each questionnaire [14]. At ages 1 and 2 years, parents reported the time (in minutes) of symptom presentation following milk consumption. At these ages, we restricted the classification of milk-related symptoms to reactions occurring ≤60 min following consumption, so as to capture the most acute symptoms that are typically associated with IgE-mediated reactions [15]. At age 16 years, children also reported on the above-mentioned reactions to milk. As there was high agreement between parent- and child-reported answers on the reactions to milk (*r* = 0.67), we based our results on parent-reported symptoms to permit alignment with questionnaires from the younger years, and to make use data on symptoms, about which parents—but not adolescents—were queried.

#### 2.2.2. Phenotypes Related to Age at Onset and Persistence of Milk-Related Symptoms

##### Persistent Milk-Related Symptoms

Symptoms in early life and/or age 8 years, and at age 16 years.

##### Transient Milk-Related Symptoms

Symptoms in early life and/or age 8 years, but not at age 16 years.

##### Adolescent Milk-Related Symptoms

No milk-related symptoms in early life or at age 8 years, but milk-related symptoms at 16 years.

#### 2.2.3. Types of Parent-Reported Symptoms at Age 16 Years

##### Symptoms Reported Specifically in Association with Milk Consumption

- Gastrointestinal: Vomiting, stomach ache/pain, diarrhoea, constipation.
- Skin: Eczema, generalised urticaria, facial oedema.
- Respiratory: Itchy, runny or stuffy nose, breathing difficulties, wheeze, dyspnoea, cough, hoarseness, indistinct speech, swollen feeling in mouth/throat.
- Cardiovascular/Neurological: Unconsciousness, pronounced fatigue.
- Anaphylaxis: Any reaction involving at least two organ systems (gastrointestinal, dermatological, lower respiratory, cardiovascular) per current criteria [16], immediately following milk consumption.

##### Symptom Reported by Children at Age 16 Years, not Specifically in Association with Milk Consumption

Recurrent abdominal pain: As defined in our previous BAMSE publication [17], recurrent abdominal pain was defined as pain, at least once per week, in the absence of a diagnosis of coeliac disease or inflammatory disease, and amongst girls, also excluding menstrual pain. In contrast to the other above-described symptoms, this was not queried specifically in relation to milk consumption, and it was answered by the children themselves.

#### 2.2.4. Other Allergy-Related Outcomes in Early Life

##### Early Life Asthma

≥3 episodes of wheezing in 12 months prior to assessments at ages 1 and/or 2, or ≥4 episodes of wheezing in the 12 months prior to assessment at age 4 years, or ≥1 episode of wheeze in combination with inhaled steroid use at any of these ages. This definition has been used in many previous BAMSE publications [13,17].

##### Early Life Eczema

Dry skin and itchy rash for ≥2 weeks, with the specific localisation of rash and/or doctor-diagnosed eczema by the age of four years.

##### Early Life Rhinitis

Suspected or evident symptoms from the nose and/or eyes after exposure to furred pets and/or pollen, and/or doctor-diagnosed allergic rhinitis by the age of four years.

#### 2.2.5. Clinical Follow-Ups: Assessment of IgE Reactivity

##### Milk IgE Reactivity

IgE antibody level > 0.35 kU_A_/L to milk performed when IgE to the food mix fx5^®^ was >0.35 kU_A_/L.

### 2.3. Statistical Analysis

Descriptive statistics (*n*, %, mean ± standard deviation, 95th percent confidence intervals (95% CI), chi-squared test, *t*-test, Fisher exact test) were used to establish possible differences between the original cohort and our study population, and between the various phenotypes and sub-populations. Statistical significance was set at *p* < 0.05. Analysis was performed with STATA Statistical Software (release 13.1; StataCorp, College Station, TX, USA).

## 3. Results

### 3.1. Baseline Characteristics

Our study population and subpopulation both corresponded well to the original cohort regarding participant characteristics, although high socio-economic status was slightly more common in the populations when compared to the original cohort (Table 1).

A total of 20.0% (537/2985) of the children had early life milk-related symptoms (Table 2). Children with symptoms had a higher prevalence of parental allergy, and, by the age of four, doctor-diagnosed food allergy, early life asthma, eczema, and rhinitis as compared to children without early life milk-related symptoms. In addition, they were first introduced to milk at a slightly older age (6.6 ± 3.2 months vs. 6.2 ± 2.5 months; *p* < 0.01). However, sex, high socio-economic status, exclusive breastfeeding for ≥4 months, and immigrant parents were comparable between the groups.

### 3.2. Prevalence of Milk-Related Symptoms from Birth to Adolescence by Age of Symptom Onset and Sex

**Figure 1:**
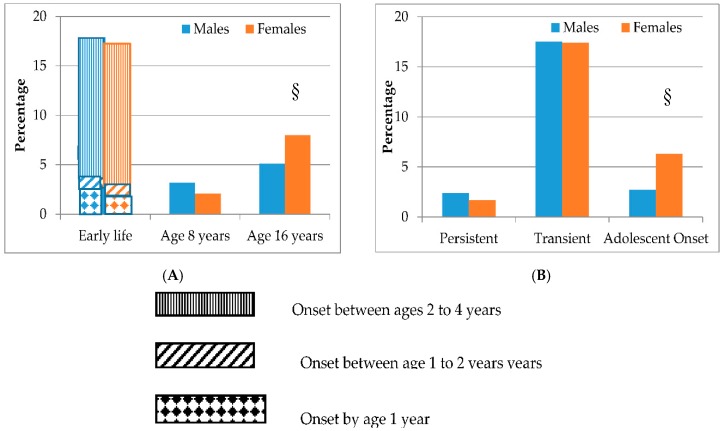
Prevalences of milk- related symptoms by gender in early life (to age 4 years) and at ages 8 and 16 years, including timing of onset (persistent *, transient † and adolescent-onset ‡) of milk- related symptoms (*N* = 2819). (**A**) Early life and ages 8 and 16 years; (**B**) Timing of onset. * Milk-related symptoms in early life and/or at age 8 years *and* at age 16 years; † Milk-related symptoms in early life or at 8 years, *but not* at age 16 years; ‡ Milk-related symptoms at 16 years, *but not* in early life or at 8 years; § *p* < 0.002 vs. the opposite sex at the corresponding time point.

The prevalence of milk-related symptoms at early life, and ages 8 and 16 years are presented in Figure 1, Panel A. The highest prevalences were seen in early life, with a comparable gender distribution (17.7% in females; 18.3% in males), and during which symptoms were most commonly presented from ages 2 to 4 years. The prevalence of symptoms decreased to age eight, affecting 2.1% of females and 3.2% of males, including 1.8% of females and 2.6% of males who had not have symptoms in early life. At age 16 years, more females than males were affected (8.0% vs. 5.1%, respectively; *p* = 0.002). With consideration to age at onset and the persistence of symptoms, the majority of children with early life symptoms had transient symptoms (94%) (Figure 1, Panel B).

Adolescent-onset symptoms were experienced by more females than males (6.3% vs. 2.7%, respectively, *p* < 0.001). Baseline characteristics and early life asthma, eczema, and rhinitis were similar between children with persistent vs. transient milk-related symptoms (Table 3). In contrast, children with adolescent-onset symptoms were more likely to be female, and were less likely to have parental allergy, doctor-diagnosed food allergy by the age of four, or early life rhinitis. Children with persistent symptoms were introduced to milk slightly later than children with transient or adolescent-onset symptoms (6.9 ± 2.9 months vs. 6.5 ± 3.2 months vs. 6.4 ± 2.4 months, respectively).

### 3.3. Types of Milk-Related Symptoms and Recurrent Abdominal Pain at Age 16 Years

Of the symptoms that were queried at age 16 years, those involving the gastrointestinal- and skin systems were most commonly reported amongst children with persistent milk-related symptoms, whereas gastrointestinal symptoms were almost exclusively reported by children with adolescent-onset symptoms (Table 4). More severe symptoms, including respiratory and/or cardiovascular/neurological systems were rare. More children with adolescent-onset symptoms report recurrent abdominal pain when compared to children with persistent symptoms (53.9% vs. 28.8%, *p* < 0.001), 22.3% with transient symptoms, and 21.4% with no reported symptoms (21.4%). Amongst children with late- onset symptoms, concomitant milk-related gastrointestinal symptoms and recurrent abdominal pain (57/134; 42.5%) and gastrointestinal symptoms in the absence of recurrent abdominal pain (48/134; 35.8%) were common, whereas recurrent abdominal pain only (11/134; 8.2%), gastrointestinal symptoms, or recurrent abdominal pain (18/134; 13.4%) were common. All of the children with milk-related symptoms at age 16 years and who responded to the question on milk consumption at 16 years (80.1% of those with symptoms) consumed milk at least once weekly (*n* = 157).

### 3.4. IgE Reactivity at Different Ages by Age of Symptom Onset

**Figure 2:**
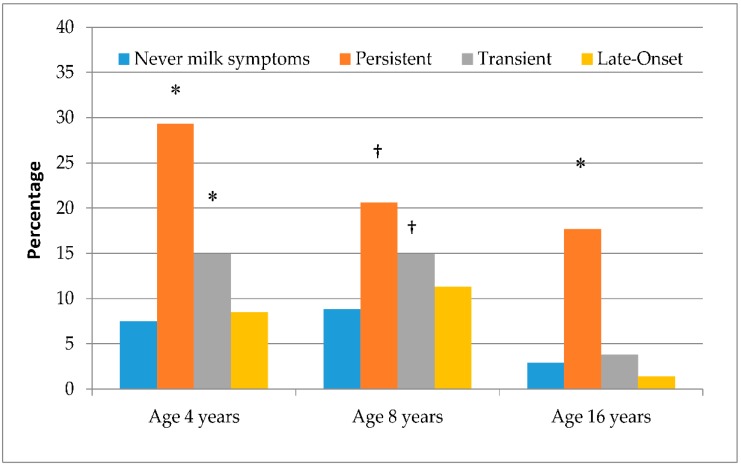
Prevalence of milk IgE reactivity (≥0.35 kU_A_/L) at ages 4, 8, and 16 years in relation to phenotype of milk-related symptoms (*N* = 1543). * *p* < 0.001 vs. never milk-related symptoms to the corresponding age; † *p* < 0.05 vs. never milk-related symptoms up to the corresponding age.

**Figure 3:**
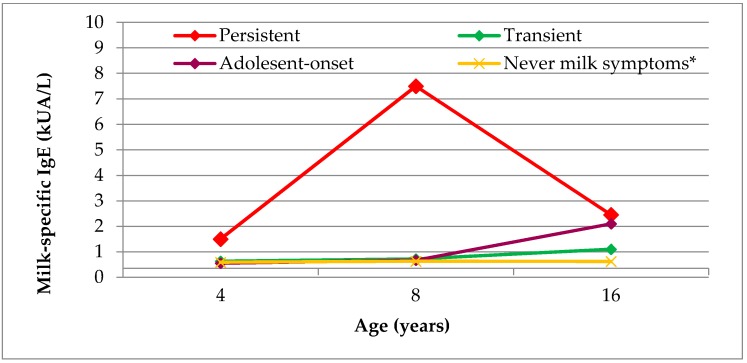
Median milk IgE levels amongst those with milk IgE reactivity (≥0.35 kU_A_/L) at ages 4, 8, and 16 years in relation to phenotype of milk-related symptoms (*N* = 1543). * Never milk symptoms to the corresponding age. Compared to mean milk IgE levels at each amongst those with persistent symptoms, all levels are significantly lower (*p* < 0.05) for those with the other phenotypes.

The prevalence of milk IgE reactivity was examined amongst children for whom sera was available at ages 4, 8, and 16 years (*n* = 1634). The highest prevalence at all ages were seen amongst children with persistent symptoms, and remained significantly higher than the prevalence of milk IgE reactivity amongst those with never milk symptoms (*p* < 0.05 for all ages; Figure 2). The proportion of children with transient symptoms and IgE reactivity did not differ statistically between ages 8 and 16 years (*p* = 0.97). Only one child with adolescent-onset symptoms had milk IgE reactivity at age 16 years (2.1 kU_A_/L). Median (range) milk IgE levels were the highest in the persistent group across all ages (age 4 years: median 11.2 (0.44-73) kU_A_/L; age 8 years: median 7.5 (0.43-64) kU_A_/L; age 16 years: median 2.5 (0.59-56) kU_A_/L; Figure 3). Amongst those with transient symptoms, milk IgE levels remained steady between ages 4 and 8 years (median: 0.53 kU_A_/L, and 0.72 kU_A_/L, respectively), but they increased by age 16 years, to 1.1 kU_A_/L. Median milk IgE levels were comparable between those with adolescent-onset symptoms and those with never symptoms to the corresponding age. As was shown in Table 4, cardiovascular/neurological symptoms at age 16 years were reported by one child with persistent symptoms and one with adolescent-onset symptoms. Milk IgE levels for these children were 56.0 kU_A_/L and 2.1 kU_A_/L, respectively. A total of 3.1% children who had never had milk-related symptoms had milk IgE reactivity at age 16 years (median: 0.62 kU_A_/L; range 0.35–5.2 kU_A_/L). 

## 4. Discussion

Amongst our population-based birth cohort of Swedish children that were followed to the age of 16, milk-related symptoms affected nearly 20% of children in early life, but were much less prevalent at ages 8 and 16 years. Most children with symptoms in early life years had transient symptoms and few reported persistent symptoms at age 16 years. The prevalence of children with milk IgE reactivity decreased from ages 4 to 16 years across all of the phenotypes. However, a greater proportion of children with persistent symptoms had milk IgE reactivity when compared to transient- or late-onset symptoms, at each age. Adolescent-onset milk-related symptoms were more common amongst females than males. Adolescents with adolescent-onset symptoms almost exclusively reported gastrointestinal symptoms after milk consumption, and milk IgE reactivity was uncommon in this group.

### 4.1. Strengths and Limitations

Our study is based on data from nearly 70% of participants in a large, population-based, unselected cohort who were followed regularly from birth to age 16 years. Repeated assessments from early life to adolescence glean insights into the age at onset and persistence of milk-related symptoms. Also, consideration to milk IgE reactivity at ages 4, 8, and 16 years revealed distinct patterns between children with persistent, transient, or adolescent-onset symptoms.

Some limitations of our study warrant mention. Milk-related symptoms were based on parental report, and thus they cannot be interpreted as milk allergy. As well, although the small sample sizes of some groups could be viewed as a limitation, we believe that this is reflective of the overall decreasing rates of milk-related symptoms beginning in late childhood, but which has not previously been studied beyond age 11–12 years [18]. Finally, as 94% of children experienced transient symptoms and only 6% experienced persistent symptoms, we were unable to analyse factors that are associated with ‘outgrowing’ milk-related symptoms.

### 4.2. Findings in Relation to Previous Studies

In keeping with previous epidemiological descriptions of symptoms that are related to milk ingestion [19], including our own cohort [20], rates of milk-related symptoms were highest in early life. However, unlike other studies in which first presentation of symptoms begins in infancy, we identified the highest rates between ages two to four years [1,3,4]. When the BAMSE children were age 0–1 year of age, in Sweden, parents of children with allergic heredity or symptoms of eczema or wheeze in infancy were advised to postpone the introduction to milk beyond the age of one year. This may explained the higher prevalence of symptoms at age two. However, at later ages, our data did not permit consideration of timing from milk consumption to symptoms. By age eight, symptoms affected less than 2%. This rate is similar to an earlier report from our cohort [20] and others [12,21]. Likewise, our finding that few adolescents had symptoms to milk parallels that of another large European birth cohort [11]. However, none of these earlier reports included results that were stratified by gender. The rates are also much lower than the estimated 15–19% of school-aged children affected by milk hypersensitivity and symptoms consistent with milk allergy elsewhere in northern Europe [5,10].

Results from the OLIN cohort from Northern Sweden showed that nearly twice as many females than males had current or outgrown symptoms (62% vs. 38%, respectively; *p* < 0.02) by age 11–12 years [10]. Although no such gender differences were found in our study for persistent and transient symptoms, approximately twice as many females than males had adolescent-onset symptoms. 

In our study, 20% of children with milk-related symptoms were IgE positive to milk. At all ages, milk IgE reactivity levels were the highest amongst children with persistent symptoms and were comparable to levels of school-aged children [4,11,21,22] and adolescents [4,11], with persistent symptoms in other industrialised nations. In contrast, the prevalence of milk IgE reactivity amongst those with transient symptoms was the highest at ages four and eight years, and decreased slightly at age 16 years. Our findings lend support to the natural history of IgE-mediated cow’s milk allergy, as described by Skripak et al. [4], wherein the milk IgE levels remain detectable subsequent to tolerance in adolescence. In the adolescent-onset symptoms group, twice as many girls as boys had adolescent-onset symptoms, which predominantly included gastrointestinal symptoms. 

Milk consumption in many high income countries, including Sweden, decreases throughout adolescence [23,24], the adolescents in our study—including those with milk-related symptoms—reported drinking milk/eating milk products once a week or more.

Milk and milk products have been described as triggers of abdominal pain in northern European populations, particularly amongst females [25,26]. In our study, nearly all children with adolescent-onset milk-related symptoms reported gastrointestinal symptoms, of whom many had recurrent abdominal pain. This latter symptom was not queried in direct relation to milk. Further, no children with adolescent-onset symptoms had detectable milk IgE reactivity. Yet, recurrent abdominal pain was twice as common amongst those with adolescent-onset vs. persistent milk-related symptoms. Herein, we did not exclude adolescents with lactose intolerance, as we were interested in reactions to milk, regardless of mechanism. Many children with lactose intolerance experience abdominal symptoms. Yet, other authors have described that less than a quarter of children with recurrent abdominal pain have clinically diagnosed lactose intolerance [27], supporting that lactose intolerance is likely not to be the primary cause of recurrent abdominal pain in our population. From a clinical perspective, it is important to note that more females than males experience adolescent-onset symptoms that are attributable to milk. Yet, such symptoms are unlikely to be caused by late-onset milk allergy, defined as having milk IgE reactivity.

## 5. Conclusions

In conclusion, milk-related symptoms are common in early life, although most children achieve tolerance by age 16 years. Milk IgE reactivity was most prevalent amongst children with persistent symptoms, but decreased in prevalence after age 8 years amongst children with transient symptoms. Adolescent-onset symptoms are more commonly reported by females, and are characterised almost exclusively by gastrointestinal symptoms, and are not associated with milk IgE reactivity.

## Figures and Tables

**Table 1:** Distribution of baseline characteristics and other allergy-related outcomes in early life of the entire cohort and the study population.

	Original Cohort (*N* = 4089)		Study Population (*N* = 2985)			Subpopulation (*N* = 1634)		
Baseline Characteristics	*n*	Percent	*n*	Percent	(95% CI)	*n*	Percent	(95% CI)
Female	2024/4089	49.5	1503/2985	50.4	48.5; 52.2	814/1634	49.8	47.4; 52.3
High socio-economic status	3323/4018	82.7	2498/2949	84.7	83.3; 86.0	1389/1616	86.0	84.2; 87.6
Exclusive breastfeeding *	3116/3919	79.5	2366/2929	80.8	79.3; 82.2	1311/1615	81.2	79.2; 83.1
Immigrant parents	718/3409	21.1	609/2979	20.4	19.0; 21.9	325/1631	19.9	18.0; 21.9
Parental allergy †	1200/4041	29.7	910/2961	30.7	29.1; 32.4	527/1621	32.5	30.2; 34.9
Doctor-diagnosed food allergy ‡	325/3299	9.0	229/2863	8.0	7.3; 9.1	130/1602	8.1	6.8; 9.6
*Other allergy-related outcomes in early life*								
Asthma	474/3629	13.1	358/2872	12.5	11.3; 13.7	204/1600	12.8	11.2; 14.5
Eczema	1272/3700	34.4	956/2895	33.0	31.3; 34.8	536/1611	33.3	31.0; 35.6
Rhinitis	545/3575	15.2	420/2817	14.9	13.6; 16.3	235/1576	14.9	13.2; 16.8

* For ≥4 months; † Parent-report of doctor-diagnosis of asthma and/or hay fever in combination with allergy to furred pets by either or both parents at time of enrollment; ‡ By age 4 years.

**Table 2:** Distribution of baseline characteristics in relation to symptoms of early life (to age four years) milk reported symptoms (*N* = 2985).

	No Early Life Milk-Related Symptoms		Early Life Milk-Related Symptoms		*p*-Value
	(*N* = 2448)		(*N* = 537)		
	*n*	Percent	*n*	Percent	
Baseline characteristics					
Females	1237	50.5	266	49.5	0.68
High socio-economic status	2049	84.7	449	84.7	0.99
Exclusive breastfeeding for ≥4 months	1945	81.0	421	79.7	0.50
Immigrant parents	488	20.0	121	22.6	0.18
Parental allergy *	709	29.2	201	37.8	<0.001
Doctor-diagnosed food allergy by age 4 years	70	3.0	159	30.3	<0.001
*Other allergy-related outcomes in early life*					
Asthma	268	11.4	90	17.2	<0.001
Eczema	668	28.2	288	54.6	<0.001
Rhinitis	266	11.6	154	29.7	<0.001

* Parent-reported doctor-diagnosed asthma and/or hay fever in combination with allergy to furred pets by either or both parents at time of enrollment.

**Table 3:** Descriptive information of participants with persistent vs. transient vs. adolescent-onset milk-related symptoms (*N* = 716).

	Persistent		Transient		Adolescent-Onset	
	*N* = 62		*N* = 520		*N* = 134	
	*n*	Percent	*n*	Percent	*n*	Percent
Baseline characteristics						
Females	26	41.9	261	50.2	94	70.2
High socio-economic status	47	75.8	438	85.4	103	78.6
Exclusive breastfeeding for ≥4 months	51	83.6	401	78.5	107	83.0
Immigrant parents	16	25.8	118	22.7	39	29.1
Parental allergy *	30	49.2	191	37.0	41	30.8
Doctor-diagnosed food allergy by age 4 years	24	40.0	141	27.8	9	7.3
*Other allergy-related outcomes in early life*						
Asthma	13	21.7	80	15.8	18	14.3
Eczema	32	53.3	276	54.1	43	33.6
Rhinitis	25	41.7	140	27.9	19	15.2

* Parent-reported doctor-diagnosed asthma and/or hayfever in combination with allergy to furred pets by either or both parents at time of enrollment.

**Table 4:** Types of parent-reported milk-related symptoms, immediately following milk consumption at age 16 years amongst adolescents with persistent vs. adolescent-onset milk-related symptoms.

	Persistent		Adolescent-Onset		*p-*Value
	(*N* = 62)		(*N* = 134)		
	*n*	%	*n*	%	
Gastrointestinal †	46	74.2	110	82.1	0.10
Skin *	15	24.2	4	3.0	<0.001
Respiratory ‡	1	1.6	2	1.5	0.68
Cardiovascular/Neurological §	1	1.6	1	0.7	0.53
Anaphylaxis	3	4.8	0	0.0	0.03

* Generalised urticaria, facial swelling, nettle rash; † Vomiting, stomach ache/pain, diarrhea; does not include recurrent abdominal pain; ‡ Itchy, runny or stuffy nose; breathing difficulties, asthma, dyspnea, cough, hoarseness, indistinct speech, itch or swollen feeling in mouth/throat; § Unconsciousness, pronounced fatigue.
